# Association between cell-free DNA fetal fraction and pregnant character: a retrospective cohort study of 27,793 maternal plasmas

**DOI:** 10.1038/s41598-023-38151-4

**Published:** 2023-07-14

**Authors:** Yaping Hou, Jiexia Yang, Fuli Deng, Fanghua Wang, Haishan Peng, Fangfang Guo, Dongmei Wang, Aihua Yin

**Affiliations:** 1 Medical Genetic Centre, Guangdong Women and Children Hospital, Guangzhou, 510010 Guangdong China; 2 Maternal and Children Metabolic-Genetic Key Laboratory, Guangdong Women and Children Hospital, Guangzhou, 510010 Guangdong China; 3 School of Food Science and Engineering, South China University of Technology, Guangzhou, 510640 China

**Keywords:** Diseases, Reproductive disorders

## Abstract

To determine the association between cell-free DNA fetal fraction (cffDNA) and various prenatal characters to better guide the clinical application of noninvasive prenatal screening (NIPS), a retrospective cohort study of 27,793 women with singleton pregnancies was conducted. Results indicated that no significant difference on cffDNA between trisomy/sex chromosome aneuploidy (SCA) and non-trisomy groups was found. However, the fetal fraction (FF) in the T18 and T13 subgroups were significantly lower than that in the non-trisomy group, while the FF in the T21 group was significantly higher than the non-trisomy group. Pearson’s correlation analysis revealed a positive correlation between √FF and gestational week in the T21, SCA, and non-trisomy groups. A negative correlation between maternal age and √FF in T21 and non-trisomy cases was found, but a positive correlation in SCA group. Compared to the decreasing trend in FF in the T21 group, no significant difference was observed in the SCA group. The √FF level was negatively correlated to maternal BMI in T21 and non-trisomy group, while a positive correlation in SCA group. FF was close related to the result of NIPS and related maternal factors. Though NIPS has increased accuracy, the complexity still should be recognized especially in clinical practice.

## Introduction

Cell-free fetal DNA (cffDNA), a very small fetal fragment (less than 200 base pairs), is thought to be derived from placental apoptotic trophoblastic cells^1,2^. It was discovered by Lo et al. in maternal plasma and serum samples in 1997^3^. On average, the cffDNA fragment is shorter than maternal cell-free DNA, and its concentration is approximately 10% of the total cell-free DNA (cfDNA) in maternal plasma^4,5^. The discovery of cffDNA in maternal plasma has accelerated the development of non-invasive prenatal testing, given that it represents a source of fetal genetic material suitable for non-invasive prenatal screening (NIPS), which has been commercially available since 2011. Although it started as a small research endeavor, it transformed prenatal care globally less than a decade after its feasibility was demonstrated^6,7^. Currently, it is available worldwide and is reported to be highly accurate for the detection of fetal chromosomal aneuploidies^6,8^. As of late 2017, about 4 million to 6 million pregnant women have undergone NIPS for fetal aneuploidy^9^. Nowadays, pregnant women are opting for safer prenatal screening, which has driven the global adoption of NIPS for chromosomal aneuploidy^10^. With the implementation of NIPS, opinion statements and recommendations have been published and updated by several academic committees throughout the world to support and guide the clinical use of NIPS in pregnant women^11–13^. Furthermore, plasma DNA-based non-invasive prenatal testing is anticipated to play an increasingly essential role in the future of obstetric care.

A wider application and a deeper understanding of cfDNA for aneuploidy detection in clinical practice by NIPS have led to the proportion of cffDNA, namely fetal fraction (FF), to be considered a key parameter affecting the performance of NIPS. Typically, a low FF may influence the efficacy of NIPS, resulting in the absence of positive findings or a “no call result” in an euploid pregnancies^10^. Hence, continuous efforts have been made to enrich cffDNA in maternal plasma during the development of NIPS. In addition, it is now evident that some maternal factors can interfere with its performance. Theoretically, any condition that promotes maternal cell turnover without increasing placental cell turnover could lower FF and increase the failure rate of NIPS. FF, which increases with advancing gestation, is influenced by various biological factors. Identifying the factors affecting FF could assist in the evaluation of FF in samples for reliable results. Despite some studies suggesting that factors such as maternal age and BMI have an impact on cffNDA, widespread consensus regarding the factors influencing the FF of cfDNA has not been reached. For instance, recent reports indicated that FF was positively correlated with gestational age and negatively correlated with maternal weight^10,14^. There are also multiple studies signaling the following: FF in average-risk pregnancies in the first trimester was not significantly different compared to FF in high-risk women^15^; maternal age was negatively related to FF^16^; FF levels increase significantly with increasing gestational age^17^. The inconsistent and contradictory results hinder the use of FF for screening. Moreover, the small sample size of the cohort study was one of the reasons for the statistical difference. Indeed, large-scale cohort studies provide more reference value.

Therefore, a retrospective cohort study summarizing 27,793 NIPS results, confirmatory invasive test results, and follow-up information of singleton pregnancies was conducted. The purpose of this study was to investigate the correlation between FF for different gestational ages, maternal weeks, BMI, and aneuploidy pregnancies, specifically fetal trisomy involving chromosomes 21 (T21), 18 (T18), and 13 (T13) and sex chromosome aneuploidy (SCA). In addition, the relationship between FF and T21, T18, T13, and SCA was evaluated.

## Methods

### Study population and sample collection

All women with singleton pregnancies who undertook NIPS at Guangdong Women and Children hospital between 20 November 2014 and 31 March 2018 were enrolled. Each participant was offered genetic counseling before and after the NIPS test, and the results were communicated to them. The study was performed under the approval of the Ethics Committee of Guangdong Women and Children Hospital (No. 201901132) and was performed in compliance with the Declaration of Helsinki. Written informed consents were obtained from each participant in the study.

For each participant, 10 mL of maternal peripheral blood sample was collected in Cell-Free DNA BCTTM tubes (Streck) and sent to the clinical laboratory within 72 h for NIPS testing. The maternal plasma was isolated by a double centrifugation procedure and stored at − 70 °C until further processing.

### Sequencing analysis of maternal plasma DNA

Cell-free DNA was extracted from 500 μL of each maternal plasma sample with the QIAamp DSP DNA Blood Mini Kit (Qiagen) following the blood and body fluid protocol. Then the DNA samples were subjected to library preparation, sequencing, and bioinformatics analysis were performed. Highthroughput sequencing of fetal-free DNA fragments was performed on an Ion Proton sequencer at 400 flows according to the manufacturer’s instructions (Life Technologies). The sequenced results of samples were analyzed blindy, and aligned to the UCSC hg19 version of the human genome using Bowtie version 2. The combined GC correction and Z score testing methods were used to identify fetal autosomal aneuploidy. Each chromosome with an absolute Z score greater than 3 was marked with chromosome aneuploidies which described our previous article for details^18^.

### Confirmatory with the diagnostic testing

Pregnant women who had a high risk of NIPT results underwent genetic counseling and were fully informed about undergoing prenatal diagnosis. Positive results of NIPS for fetal aneuploidies were identified via karyotype analysis (the resolution of G-banding was 400 bands) or chromosomal microarray analysis (CMA) (CytoScanTM 750K, available from Affymetrix, USA). The karyotype analysis was performed on fetal DNA, cultured ammiocytes or lymphocyte under sterile conditions according to standard protocols, and fetal genomic DNA was also extracted from amniotic fluid or cord blood according to the manufacturer’s protocol of CMA. Thus, the NIPS result could be compared to the gold standard diagnostic test result. And these testings were also performed for all follow-up confirmatory diagnostic testing via telephone interviews to examine the placenta postnatally in cases of false-positive results.

### Statistical analysis

All data were presented as Means ± Standard Deviation (M ± SD). The statistical software package SPSS 13.0 was used for statistical analyses. Statistical methods were used to evaluate the correlation between fetal fraction and maternal characteristics of chromosomes 21, 18 and 13. The measured fetal fraction was represented as square root (√) transformed distribution to ensure the normality as described earlier. The association between fetal fraction and maternal characters was calculated by Pearson’s correlation analysis. The differences among levels of variables were compared pairwise using one-way analysis of variance test with post hoc Tukey's HSD (honest significant differences) test. *P*-value < 0.05 indicated a statistically significant difference. Relationships between fetal DNA fraction and maternal characteristics including maternal week, maternal age and BMI in chromosomes 21, 18 and 13 were demonstrated as scatter plots. The statistical analyses, except the trend test, were performed using statistical software package SPSS 22.0 (SPSS, Chicago, IL, USA). The Pearson’s correlation analysis was performed using R version 2.13.0, EZR on R commander version 1.1 designed to add statistical functions frequently used in biostatistics^19,20^. Based on the binary hypothesis Z-score evaluation criteria, the risk probability for the targeted chromosomes 21, 18, 13 and sex chromosome is statistically analyzed by the special non-invasive prenatal data analysis management system software.

### Ethics approval and consent to participate

This work was approved by the Ethics Committee of Guangdong Women and Children Hospital, Guangzhou, Guangdong 510010, China. And the written informed consents were obtained from all the participants in present study.

## Results

### Dataset summary of the study population

27,793 singleton pregnant subjects underwent NIPS at Guangdong Women and Children hospital from November 20, 2014, to March 31, 2018 were enrolled. The FF level and baseline characteristics of the pregnant women who participated in the study were presented in Table 1.

**Table 1:** Summary of the fetal fraction and characteristics of the study population.

	Median	Std.deviation	Minimum	Maximum
Fetal fraction (%)	13.46	5.10	4.01	42.48
Gestational age (weeks)	17.00	4.13	12.00	36.00
Maternal BMI (kg/m^2^)	22.00	3.13	14.00	41.00
Maternal age (years)	31.00	5.46	18.00	48.00

*BMI* body mass index.

**Figure 1:**
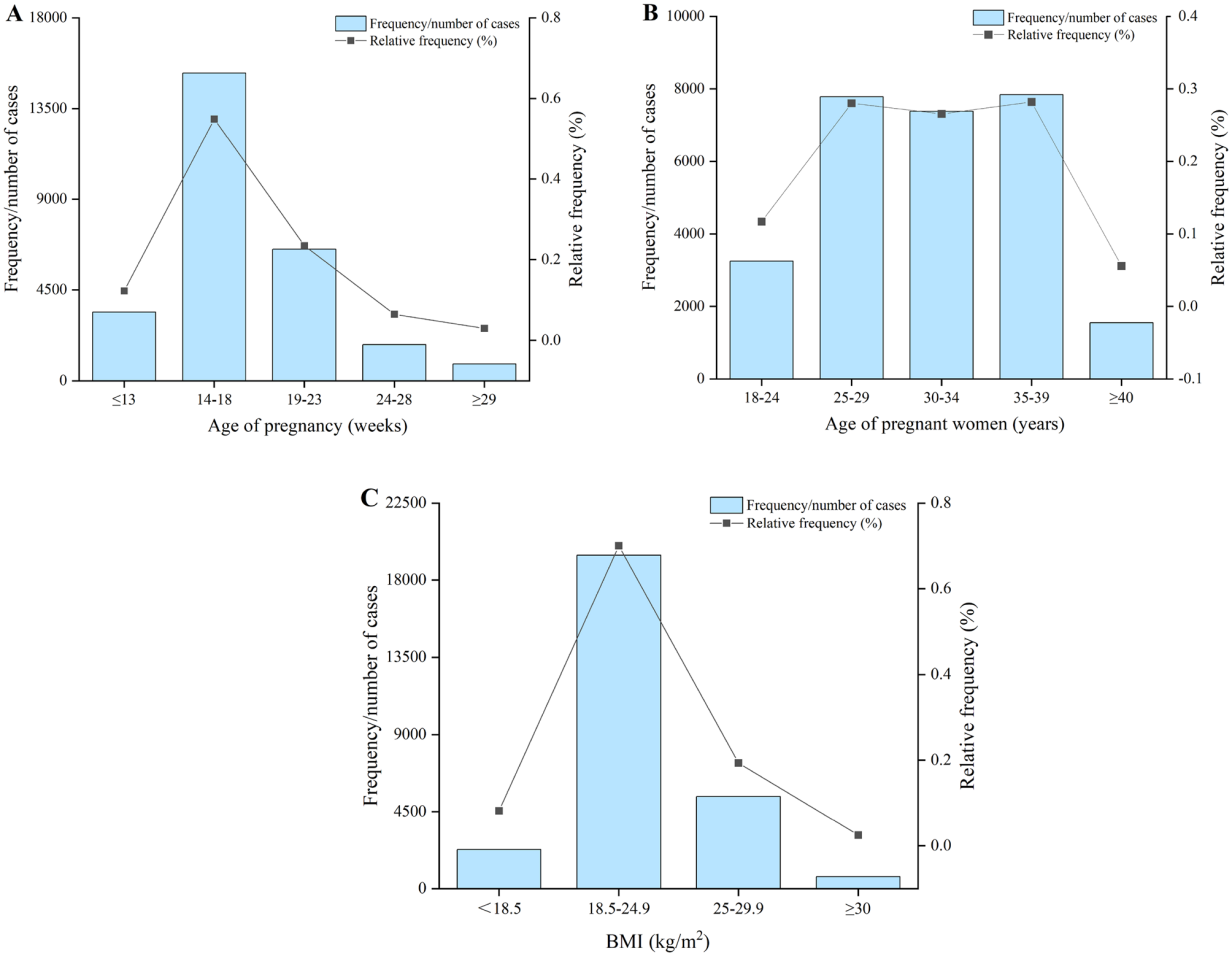
Distribution of the different factors for the 27,793 pregnant subjects in this study. (**A**) Distribution of the gestational age of the 27,793 subjects. (**B**) Distribution of the maternal age of the 27,793 subjects. (**C**) Distribution of the BMI of the 27,793 subjects.

The gestational age at sampling for NIPS was between 14 and 18 weeks (15,255, 54.89%) for more than half of the 27,793 eligible subjects; the remaining subjects (12,538, 45.11%) belonged to four different gestational age groups, among them, nearly a quarter of them were in the 19–23 weeks group (6515, 23.44%), and then were belong to the ≤ 13 group (3401, 12.23%) (Fig. 1A). In addition, more than a quarter of them belonged to the 25–29 years (7784, 28.00%), 30–34 years (7377, 26.54%), and 35–39 years (7837, 28.20%) age groups. The remaining were in the 18–24 years and ≥ 40 years age groups (Fig. 1B). Despite the small size of the different groups, around 69.97% of the cases were within the maternal BMI range of 18.5–24.9 kg/m^2^ (Fig. 1C).

Of the 27,793 cases, 217 common autosomal trisomies (T21, T18, and T13) cases (0.78%) were detected by NIPS; 135 cases were T21, 49 cases were T18, and 33 cases were T13. Moreover, there were 124 positive cases (0.45%) of SCA. Invasive prenatal diagnosis confirmed 101 cases as T21, 28 cases as T18, 10 cases as T13, and 43 cases as SCA. Furthermore, 2 negative-cases which were detected by NIPS were further confirmed as T18 via invasive prenatal diagnosis. The positive predictive value (PPV) was 82.11%, 66.67%, 31.25%, and 50.59%, respectively, while the negative predictive value was 100%; the false positive rate (FPR) was 0.02%, 0.02%, and 0.02%, respectively. The sensitivity rate and the specificity rate of NIPS for T21, T18, T13, and SCA were 100%, 93.33%, 100%, 100% and 99.92%, 99.95%, 99.92%, and 99.85%, respectively; the false positive rate and the false negative rate of NIPS for T21, T18, T13, and SCA were 0.08%, 0.05%, 0.08%, 0.15% and 0%, 6.67%, 0%, 0%, respectively.

### Relationship between fetal fraction and different trisomy results via NIPS

**Figure 2:**
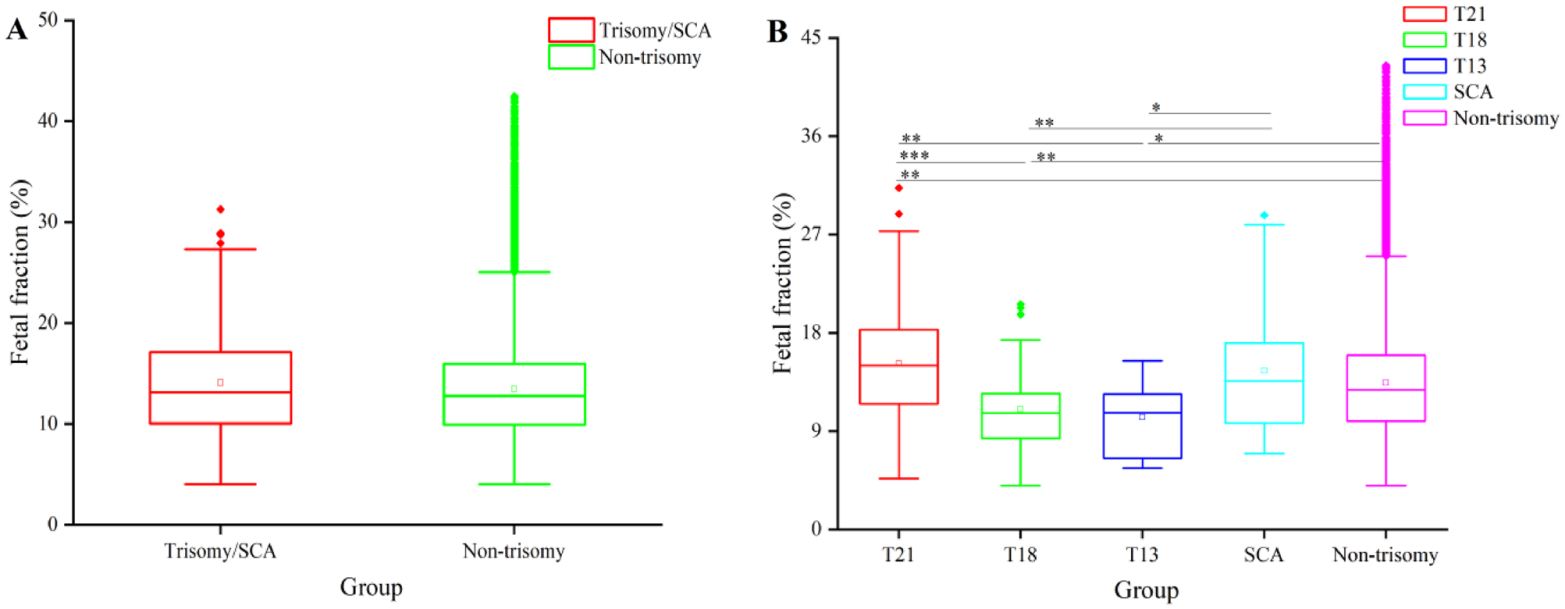
Effects of different trisomies on the fetal fraction in pregnant subjects. (**A**) Fetal fraction in the Trisomy/SCA and Non-trisomy groups. (**B**) Fetal fraction in the subjects carrying fetuses with different trisomies. *P < 0.05; **P < 0.01; ***P < 0.001.

Figure 2 illustrates the FF levels of pregnant women carrying fetuses with different trisomies. The data revealed that the FF level in the trisomy/SCA group increased to some extent, but there was no significant difference between the trisomy/SCA and the non-trisomy groups (Fig. 2A).

The subjects in the trisomy/SCA group were further subdivided into four groups: the T21, T18, T13, and SCA groups. Although the mean FF in non-trisomy group as per NIPS was 13.45% (n = 27,550), the mean FF level for T21 (n = 101), T18 (n = 30), T13 (n = 10) and SCA (n = 43) were 15.18%, 11.03%, 10.27% and 14.56%, respectively (Fig. 2B). The FF in the T18 (*P* < 0.01) and T13 (*P* < 0.05) groups were significantly lower than that in the non-trisomy group. In contrast, the FF in the T21 group (*P* < 0.01) was significantly higher than in the non-trisomy group. Additionally, the FF level was significantly lower in the T18 (*P* < 0.001) and the T13 groups (*P* < 0.01) compared to the T21 group. Lastly, the FF in the T18 (*P* < 0.01) and the T13 (*P* < 0.05) groups were significantly lower than that in the SCA group (Fig. 2B).

### Correlation between gestational week and fetal fraction in the different trisomy groups detected by NIPS

**Figure 3:**
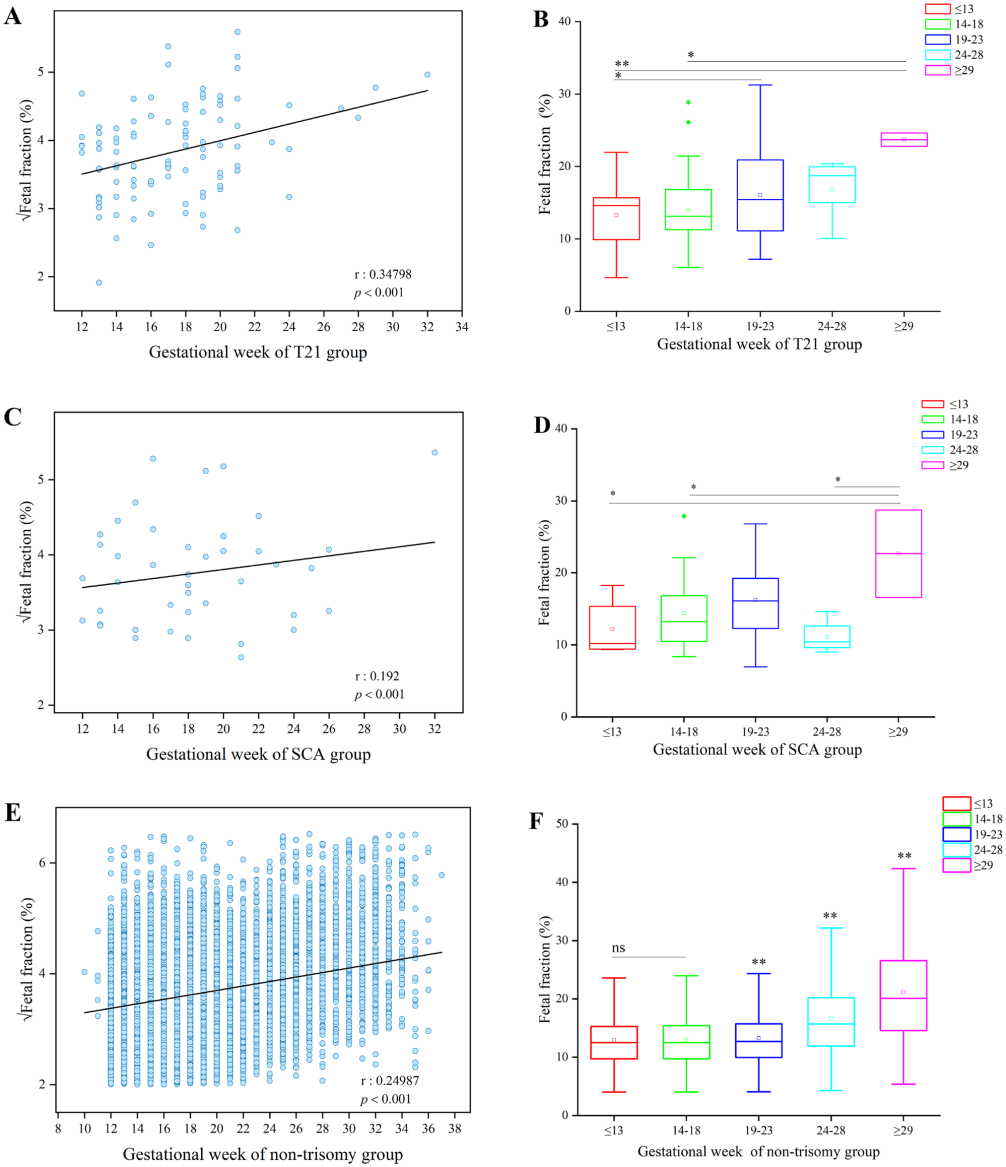
Effect of gestational week on fetal fraction and correlation between fetal fraction and gestational week in the different trisomy and non-trisomy groups detected via NIPS. (**A**) Gestational week was positively correlated with √fetal fraction in the T21 group, as detected by NIPS. (**B**) Effect of gestational week on fetal fraction in T21 as detected by NIPS. (**C**) Gestational week was positively correlated with √fetal fraction in SCA as detected by NIPS. (**D**) Effect of gestational week on fetal fraction in SCA as detected by NIPS. (**E**) Gestational week was positively correlated with √fetal fraction in non-trisomy cases. (**F**) Effect of gestational week on fetal fraction in non-trisomy cases. *P < 0.05; **P < 0.01; *ns* no statistical significance.

Pearson’s correlation analysis revealed a positive correlation between √FF and gestational week in the T21, SCA, and non-trisomy groups (Fig. 3A,C,E). In the T21 group, compared with the ≤ 13 weeks group, the FF level was significantly increased in the 19–23 weeks group (*P* < 0.05) and the ≥ 29 weeks group (*P* < 0.01). Besides, FF in the ≥ 29 weeks group was higher than in the 14–18 weeks group (*P* < 0.05) (Fig. 3B).

In the SCA group, the fetal fraction level was significantly decreased in the ≤ 13 weeks group, the 14–18 weeks group, and the 24–28 weeks group compared with the ≥ 29 weeks group, respectively (*P* < 0.05) (Fig. 3D). In contrast, there were no significant differences between gestational week and FF in either the T18 or T13 group for the small number of cases. Meanwhile, the FF in non-trisomy cases increased with gestational week, with the highest FF in the ≥ 29 weeks group (*P* < 0.05) (Fig. 3F).

### Relationship between maternal age and fetal fraction in the different trisomy groups as detected by NIPS

**Figure 4:**
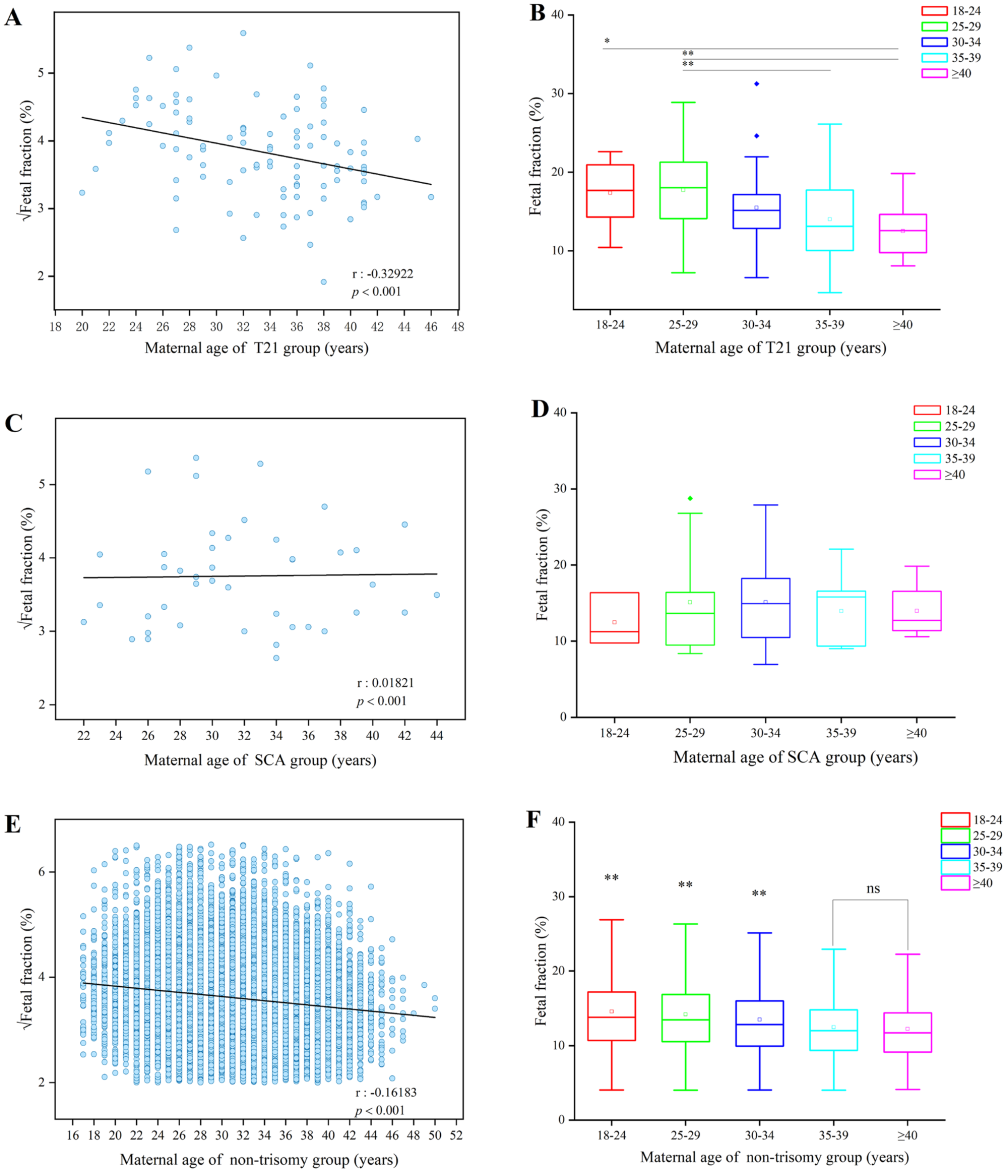
Effect of maternal age on fetal fraction and correlation between fetal fraction and maternal age in the different trisomy and non-trisomy groups as detected by NIPS. (**A**) Maternal age was negatively correlated with √fetal fraction in the T21 group as detected by NIPS. (**B**) Effect of maternal age on fetal fraction in the T21 group as detected by NIPS. (**C**) Maternal age was positively correlated with √fetal fraction in the SCA group as detected by NIPS. (**D**) Effect of maternal age on fetal fraction in the SCA group as detected by NIPS. (**E**) Maternal age was negatively correlated with √fetal fraction in non-trisomy cases. (**F**) Effect of maternal age on fetal fraction in non-trisomy cases. *P < 0.05; **P < 0.01; ns, no statistical significance.

Except for gestational week, maternal age was also related to FF level. The results revealed a negative correlation between maternal age and √FF in T21 and non-trisomy cases, but a positive correlation in SCA group (Fig. 4A,C,E). In the T21 group, the FF level in the ≥ 40 years age group was significantly lower than that in the 18–24 (*P* < 0.05) or 25–29 years age groups (*P* < 0.01). Compared to the 25–29 years age group, the level of FF was significantly lower in the 35–39 years age group (*P* < 0.01). There was a decreasing FF trend in the T21 group (Fig. 4B). Compared to the change in FF in the T21 group, the overall FF trend was more stable, and there was no significant difference in the SCA group (Fig. 4D). The results for the non-trisomy group were consistent with those of the correlation analysis (Fig. 4F).

### Relationship between maternal BMI and fetal fraction in the different trisomy groups as detected by NIPS

**Figure 5:**
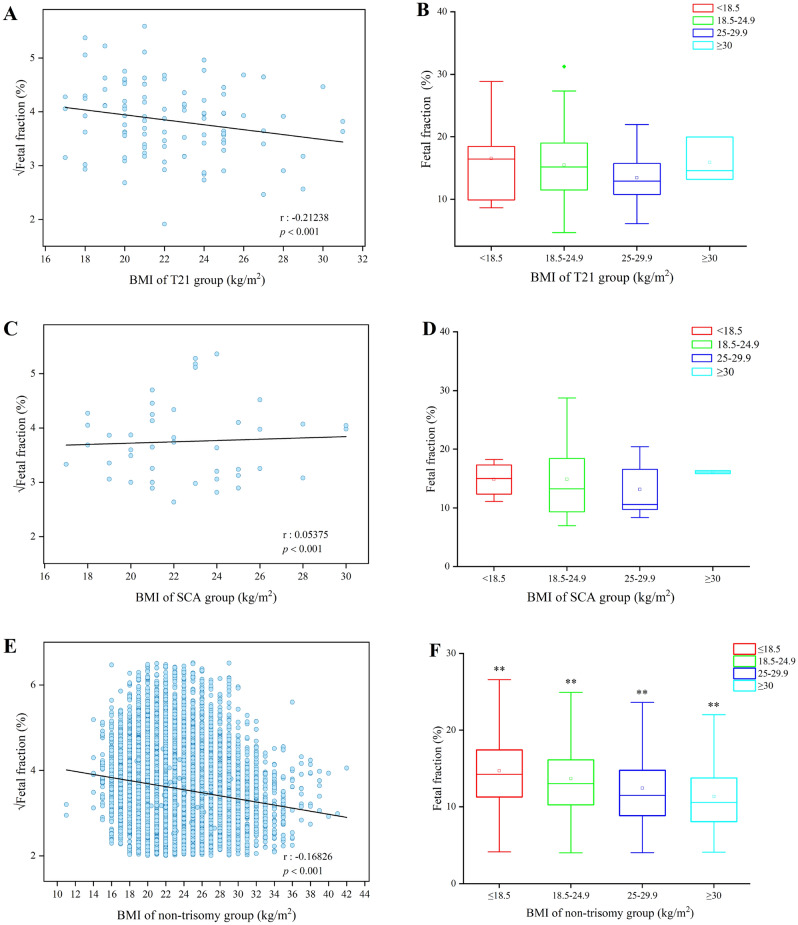
Effect of BMI on fetal fraction and correlation between fetal fraction and BMI in the different trisomy and non-trisomy groups as detected by NIPS. (**A**) BMI was negatively correlated with √fetal fraction in the T21 group as detected by NIPS. (**B**) Effect of BMI on fetal fraction in the T21 group as detected by NIPS. (**C**) BMI was positively correlated with √fetal fraction in the SCA group as detected by NIPS. (**D**) Effect of BMI on fetal fraction in the SCA group as detected by NIPS. (**E**) BMI was negatively correlated with √fetal fraction in non-trisomy cases. (**F**) Effect of BMI on fetal fraction in non-trisomy cases. *P < 0.05; **P < 0.01.

Body mass index (BMI), calculated from weight and height, was used to objectively divide the subjects into four groups based on the World Health Organization obesity classification system. The results indicated that the √FF level negatively correlated with maternal BMI in T21 and non-trisomy group, while a positive correlation in SCA group (Fig. 5A,C,E). With the exception of the ≥ 30 kg/m^2^ group, compared with the < 18.5 kg/m^2^ group, there was a decreasing trend in the 18.5–24.9 kg/m^2^ group and 25–29.9 kg/m^2^ group, respectively either in the T21 group or SCA group (Fig. 5B,D), and non-trisomy group demonstrating a steady downward trend (Fig. 5F). The results showed a decrease in FF with increasing maternal BMI. However, there was no significant difference within the subgroups in T21 and SCA group.

## Discussion

Although NIPS has higher accuracy than conventional prenatal screening methods, challenges regarding its clinical implementation, including test failure, false positive and false negative rates, findings of unclear significance, and potential maternal health implications of abnormal results, have become more apparent with a deeper understanding and wider application of this technology^10^. In the present research, 27,793 pregnant women who underwent NIPS were included, and the relationships between the fetal fraction, trisomy/SCA, and maternal factors were explored. This study contributed a large-scale clinical dataset to the prenatal screening field. Approximately 3% to 5% of pregnancies are complicated by birth defects or genetic disorders. Chromosomal abnormalities, including T21, T18, T13, and SCA, are present in approximately 1 in 150 live births^21–24^. Recently, cffDNA, a potential source of fetal genetic material, has become available for NIPS. NIPS has been extensively used, especially for T21, T18, T13, and fetal aneuploidy screening following the discovery of cffDNA^3^ and the development of the genetic testing technique^2,20,25–27^.

Since cffDNA arises from placental apoptotic trophoblastic cells, the awareness of the screening nature and limitations of the technology should be increased. The fetal fraction level was calculated for quality control using our previously described method^28^. The fetal fraction level was determined from the proportion of Y-chromosome sequences in maternal plasma. The estimated fetal fraction of a sample including female and euploid fetuses was calculated by the correlation to the equation. cffDNA is an important quality metric for NIPS, and exploring factors affecting FF is meaningful for its clinical application^29^. The present study indicated that there was no difference in FF between the trisomy/SCA and non-trisomy groups (Fig. 2A). When the trisomy/SCA group was further divided into T21, T18, T13, and SCA groups, the FF in the T18 (*P* < 0.01) and the T13 groups (*P* < 0.05) was significantly lower compared to the non-trisomy group. However, the FF in the T21 (*P* < 0.01) group was significantly higher than that in the non-trisomy group (Fig. 2B). Meanwhile, the FF in the T18 (*P* < 0.01) and the T13 (*P* < 0.05) groups was significantly lower than that in the SCA group (Fig. 2B). Even though there exists an increasing trend in the SCA group compared with the non-trisomy group, it was not statistically significant. This study demonstrated that chromosome aneuploidy could affect FF, consistent with previous studies^30^. According to earlier studies, both the low FF in T18/T13 and the high FF in T21 could be one of the reasons behind the performance of NIPS for T21 being superior to that of T18/T13^21,30^. Therefore, this study uncovered not only the complexity of the factors affecting fetal fraction but also the screening performance of NIPS.

The results of this large-scale clinical study of 27,793 pregnant women indicated that the level of FF in patients undergoing NIPS for different trisomies could be affected by gestational age, maternal age, or maternal BMI, especially for T21 or SCA. According to the results, the level of fetal fraction showed an overall increasing trend in both the T21 and the SCA group. Notably, the level of FF in the T21 groups increased incrementally from 19 weeks of gestation (Fig. 3A). More importantly, there was a significant decrease in FF in the ≤ 13 weeks, 14–18 weeks, or 24–28 weeks groups compared to the ≥ 29 weeks group (*P* < 0.05) in the SCA cases (Fig. 3B). In line with the findings of previous studies, FF in maternal blood rose with gestational age^23,24^; FF increased incrementally between 10 and 21 weeks of gestation and over this gestational age window^12,26^. Therefore, this study determined that gestational age was one of the factors that affected the increase in FF levels in the trisomy cases detected by NIPS. Also, there were no significant differences between gestational age and FF in the T18 and the T13 groups for the small number of cases in some groups. Research to gather more concrete data in this area is ongoing.

In women with high BMI, FF tended to be lower, possibly attributable to higher amounts of maternal cfDNA from maternal adipose tissue apoptosis^6,19,31^. Moreover, maternal age was linked to the FF level in NIPS-positive cases. Our results showed that maternal age was also related to FF, especially in the T21 group (Fig. 4). Consistent with previous research results^32^, this study validated that FF decreased as maternal age increased (Fig. 4A). Maternal age or other factors for aneuploidy could affect the performance of prenatal screening methods using cfDNA^26^. However, there was no significant trend between the maternal age and FF, such as in the SCA, the T18, and the T13 groups. This study also signaled that FF was not correlated with maternal age^27^. The other reason was from the study population: In China, the practice of late marriage and late childbirth has been implemented for several years. Thus, most of the pregnant women in this study were 25–39 years old, and nearly one-third of them were 35–39 years old (Fig. 1B). Invasive prenatal diagnosis was recommended for pregnant women aged 35 years or older. Overall, these national conditions and policies generated an uneven distribution of the age of the pregnant women. However, no consensus has been reached on the impact of maternal age on fetal fraction, and concrete data are warranted to confirm whether maternal age affects fetal fraction.

Except for gestational age and maternal age, the outcomes of this study implied that body mass index (BMI) could be associated with the FF level in different trisomic pregnancies undergoing NIPS. BMI, calculated using weight and height, may be more descriptive of maternal physiologic conditions than weight alone. Therefore, it was used to objectively divide the eligible subjects into four groups according to the World Health Organization obesity classification system. Regarding the subjects in both the T21 and SCA groups, the results showed that the fetal fraction level could be negatively correlated with maternal BMI. Even though there seemed to be some differences related to the maternal BMI in the T21 or the SCA groups, there were no significant differences between the positive group and BMI (Fig. 5). Besides, owing to limited T18 or T13 cases, there was no significant difference in either of these groups. These results were inconsistent with earlier studies on the association of BMI and FF^29,33,34^. There could be two reasons for these discrepancies. To begin, there were some differences in research objectives. Compared with other studies, the present one aimed to analyze the differences between different maternal BMI in certain positive groups. The few positive subjects in the different groups could also have affected the trend of the results. To the best of our knowledge, no similar studies have been reported. However, in the future, the accumulation of the positive cases for the small groups will still be required. The second cause could be that FF was associated with numerous factors, such as gestational age, maternal BMI, maternal diseases, and experimental method. For instance, there was a significant positive correlation between FF and gestational age, concordant with previously reported findings^29,32,34^.

This study presented useful clinical data regarding the utility of NIPS and diagnostic testing. However, this study had some limitations that need to be taken into account. First, the relationship between FF and other factors, such as the free β-hCG and PAPP-A levels, was not evaluated. Second, this study lacked clinical data on maternal conditions, such as blood glucose and lipid levels, and other potential confounding characteristics, such as smoking, alcohol intake, and dietary habits. Other factors could have affected FF levels and/or the accuracy of NIPS. Thus, further investigation and clinical studies are warranted.

## Conclusions

The present study provided meaningful clinical data on NIPS results. The findings herein indicated that the FF level in maternal plasma is related to the NIPS results, such as T21, T18, T13, and SCA. The FF in the T21 group was significantly higher than that in the non-trisomy group. However, the results showed that T18 and T13 could significantly affect the decrease in FF. Nevertheless, there was some increase in the FF level in the SCA samples compared to the non-trisomy samples. In addition, the results signaled that the FF level in maternal plasma was correlated to varying degrees to numerous factors, such as gestational age, maternal age, and maternal BMI. In conclusion, it is well recognized that the FF plays a key role in the performance of NIPS for diagnosing autosomal aneuploidies. However, FF could be influenced by various factors. Therefore, although NIPS is better understood and utilized for its high safety and efficiency, the complexity and limitations should still be addressed, particularly in clinical practice.

## Data Availability

The dataset generated and analyzed during the current study are not publicly available. An anonymized version of the dataset is available from corresponding author of the study (A.Y.) on reasonable request.

## Abbreviations

cffDNA: Cell-free fetal DNA
NIPS: Noninvasive prenatal screening
FF: Fetal fraction
√FF: Square root (√) of fetal fraction
cfDNA: Cell-free DNA
SCA: Sex chromosome aneuploidy
PPV: Positive predictive value
FPR: False positive rate
BMI: Body mass index
